# Mothra: Multiobjective *de novo* Molecular Generation Using Monte Carlo Tree Search

**DOI:** 10.1021/acs.jcim.4c00759

**Published:** 2024-09-25

**Authors:** Takamasa Suzuki, Dian Ma, Nobuaki Yasuo, Masakazu Sekijima

**Affiliations:** † Department of Computer Science, Tokyo Institute of Technology, Yokohama, Kanagawa 226-8501Japan; ‡ Tokyo Tech Academy for Convergence of Materials and Informatics (TAC-MI), Tokyo Institute of Technology, Tokyo 152-8550, Japan

## Abstract

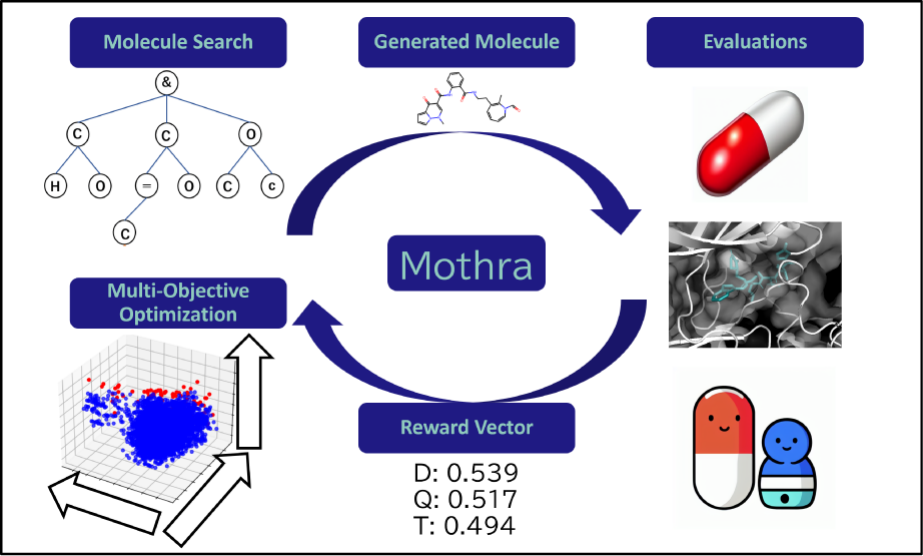

In the field of drug discovery, identifying compounds that satisfy multiple criteria, such as target protein affinity, pharmacokinetics, and membrane permeability, is challenging because of the vast chemical space. Until now, multiobjective optimization via generative models has often involved linear combinations of different reward functions. Linear combinations solve multiobjective optimization problems by turning multiobjective optimization into a single-objective task and causing problems with weighting for each objective. Herein, we propose a scalable multiobjective molecular generative model developed using deep learning techniques. This model integrates the capabilities of recurrent neural networks for molecular generation and Pareto multiobjective Monte Carlo tree search to determine the optimal search direction. Through this integration, our model can generate compounds using enhanced evaluation functions that include important aspects like target protein affinity, drug similarity, and toxicity. The proposed model addresses the limitations of previous linear combination methods, and its effectiveness is demonstrated via extensive experimentation. The improvements achieved in the evaluation metrics underscore the potential utility of our approach toward drug discovery applications. In addition, we provide the source code for our model such that researchers can easily access and use our framework in their own investigations. The source code and pretrained model for Mothra, developed in this study, along with the Docker image for the Pareto front explorer and compound picker, designed to streamline the selection and visualization of optimal chemical compounds, are released under the GNU General Public License v3.0 and available at https://github.com/sekijima-lab/Mothra.

## Introduction

Research and development before a new drug is generally approved for clinical use is a long, expensive, and challenging process, taking 12–15 years and an average of $2.6 billion.^1,2^ In general, the process of drug development is a process of target validation, compound screening, lead optimization, preclinical testing, phases I, II, and III, and approval for launch. The failure rate of drug development exceeds 90% when considering both preclinical candidates and clinical trials.^3−5^ 90% of these failures are due to deficiencies in drug candidates in terms of factors like clinical efficacy, toxicity, and drug-like properties, and it is expected that mitigating these problems leads to more successful drug discovery.^4,6^ The number of new drugs approved in recent years has not increased compared to the number of newly discovered compounds. Therefore, a compound used as a drug must satisfy multiple criteria. The conditions described above illustrate the difficulty of discovering compounds with appropriate absorption, distribution, metabolism, excretion, and toxicity (ADMET) profiles as drugs, in addition to considering their binding affinities to drug target proteins, in a chemical space estimated to contain approximately 10^60^ different compounds.^7,8^ In summary, a drug candidate must meet multiple requirements.

High-throughput screening (HTS) is widely used as a first step in drug discovery. However, because HTS is applied to known compound libraries, it is difficult to cover the entire vast chemical space. This limitation underscores the need for new discovery methods and techniques. With recent advances in computers and algorithms, the application of computer technologies to drug discovery has been explored, leading to improvements in the efficiency and quality of the drug discovery process.^9−14^ High-throughput virtual screening is widely used to search hit molecules in in-silico drug discovery^15−17^ however, the aforementioned limitation remains. To expand the chemical space without relying on existing compound libraries, deep-learning-based methods called molecular generative models have emerged, such as the VAE-based^18,19^ GAN-based,^20^ genetic-algorithm-based,^21^ and reinforcement learning^22,23^ -based methods. These models have explicit or implicit objective functions. During training, they maximize or minimize their objective functions to optimize the functions. Optimization problems often feature a single-objective function under several constraints. The formulation of optimization problems predominantly incorporates a single objective function in the realm of in silico drug discovery. For example, ChemTS^24^ is a normalized combined function of the octanol–water partition coefficient logP, synthetic accessibility score (SAscore),^25^ and ring penalty. Additionally, SBMolGen^26^ employs a normalized function of the docking score. Moreover, many other indicators are used in in-silico drug discovery. The quantitative estimate of drug-likeness (QED)^27^ captures the abstract notion of aesthetics in medicinal chemistry based on Lipinski’s rule of five.^28^ When the QED is high (up to 1), the molecule is estimated to be a drug-like compound from the viewpoint of physical chemistry. Recently, the F*sp*^329^ indicator was proposed for HTS. As the drug discovery stage progresses, the ratio of carbon atoms with *sp*^3^ electrons increases. However, molecules with higher F*sp*^3^ scores are more likely to contain chiral carbon atoms. Many chiral carbon atoms increase the 3D structural activity but decrease the synthetic feasibility. Other indicators that should be optimized include membrane permeability, molecular weight, aqueous solubility, and metabolic stability. In summary, many metrics of drug performance and safety should be considered during in-silico drug discovery.

Conceptually, an optimizer for multiple objective functions is significantly different from that of a single function. In actual drug discovery, it is necessary to simultaneously optimize several situation-specific physical property indicators. However, simultaneously optimizing multiple objective functions results in the problem of Pareto optimality. Some recently published studies avoided the problem of Pareto optimality by converting multiple evaluation indices into a single evaluation function using linear summation^30^ or multiplier-adjusted multiplication.^31,32^ These methods are based on the same optimizing strategy of desirability score (DScore). However, these methods have the common problem of adjusting the weights of the evaluation indices. For example, a QED score of “0.5” should differ from a docking score of “0.5”. Additionally, assigning weights to the evaluation indices requires comparing the values in different objective functions. the weighted sum is not suited. In manufacturing, the cost and risk of the process are trade-offs that cannot be added together for maximization or minimization.^33^ Li et al.^34^ constructed a multiobjective *de novo* drug design system with a conditional variational autoencoder (CVAE);^35^ however, this method cannot explicitly handle multiple objective functions. Reutlinger et al.^36^ introduced the Gaussian process for regression^37^ to multiobjective optimization (MOO). However, this method cannot handle explicit objective functions.

Simultaneous optimization of multiple objectives is a common challenge in drug development. For example, many metrics with different objectives, such as drug efficacy, safety, and production costs, must be optimized simultaneously. Because these objectives are often in a trade-off, optimizing one objective can worsen the others. MOO with a Pareto front has been proposed as a general approach for solving such multiobjective-function optimization problems. The Pareto front represents the set of solutions for which all objective functions are optimal, with the property that no solution on the front can improve any other objective without worsening any of the objectives. Therefore, the Pareto front can be used to determine the optimal solution while considering multiple objective functions simultaneously. Recent methods in MOO considering the Pareto frontier have been proposed using the genetic algorithm (GA).^38−41^ GA-based algorithms generally search for molecules by evaluating their hidden vectors. Thus, GA-based algorithms make it difficult to obtain the information on generated molecules in a search. However, Monte Carlo tree search (MCTS) contains a simulation step. In the simulation step on each search epoch, the scores calculated on the basis of the generated molecules are fed back to the search tree. MCTS obtains the information on generated molecules during the search. Thus, the MCTS method is suitable for modifying generated molecules because the search tree enables the search for structurally similar molecules by fixing the heads of molecules in SMILES strings.

Molecules can be represented in a variety of ways, including chemical fingerprinting techniques such as extended-connectivity fingerprints (ECFPs),^42^ which use fixed vectors for different substructures, and the simplified molecular input line entry system (SMILES),^43^ which represents molecules in a string format. In particular, SMILES can accurately capture structural variations, including the chirality of compounds. However, the use of SMILES in deep learning-based molecule generation models presents unique challenges, particularly in the hit-to-lead process. Generating molecules from predefined structures remains a significant challenge, and most SMILES-based methods cannot reliably generate valid molecules from a starting point. However, a new approach presented in MERMAID^44^ combines a Monte Carlo tree search and recurrent neural networks to introduce a SMILES-based generative model that can start from a specific molecule.

In this research, we developed a *de novo* molecular generation model for easily extendable multiple objective functions using SMILES via Pareto-based multiobjective MCTS. To evaluate the molecule, we set the docking score using SBMolGen,^26^ QED score, and estimated toxicity probability^45^ as reward functions. Additionally, the SAscore^25^ was used as a thresholding for highly difficult-to-synthesize molecules. However, in more practical and precise cases, if users have knowledge of organic chemistry and know the substructures that they want to avoid, they can implement filters based on the substructures of the method. The simulator used to calculate the docking score was AutoDock Vina.^46^ The proposed method succeeded in generating multiobjective optimized molecules in a target protein and is available on GitHub. Furthermore, the viewer available in the same repository on GitHub enables users to dive into the chemical space. The existing methods often rank generated molecules. The act cannot capture the trade-off relationship. The viewer provides the dots corresponding to the generated molecules, such as the following figures in this paper and shows the molecular structures and docking poses. With the viewer, users easily access the chemical space only by clicking.

## Method

### Mothra Overview

Mothra is a Pareto Monte Carlo tree search-based molecular optimizer. As a structure generator, we used both the recurrent neural network (RNN)^47^-based structure generator installed in both ChemTS^24^ and MERMAID^44^ and a multiobjective Monte Carlo tree search (MOMCTS)^33^ -based exploration system. ChemTS is a prototype of the molecular generator. To direct the exploration without calculating the DScore, that is, without adjusting the weights, we employed Pareto optimization. Pygmo^48^ based on NSGA-II^49^ was applied to estimate whether the molecule was in the Pareto front or not. The Monte Carlo tree search (MCTS) has four steps in every search. Using the first two steps, Mothra searches for and determines the heads of the molecules. In the last two steps, Mothra generates and evaluates molecules, followed by a series of evaluations in an MOO framework, which can be extended to accommodate drug design requirements.

**Selection**: Each node in the search tree contains one character in the SMILES^43^ vocabulary, which may represent an element or a structure. In the selection step, an expandable node is selected through the tree policy while considering the Pareto front. The path from the root node to the selected (leaf) node is a substring at the beginning of the SMILES-represented molecules currently being searched.
**Expansion**: One node is added to the search tree as a child node of the selected node. Thus, the addition of this node adds one character in the SMILES grammar to the end of the substring obtained in the selection step.
**Simulation**: The pretrained RNN acts as the default policy to complement the molecule in the simulation step. After completing the SMILES string, the string is checked to express a valid molecule. The molecules are evaluated to obtain reward vectors, each of which is compared with each vector in the Pareto front to determine whether it is a dominant vector or not.
**Backpropagation**: The reward vectors are fed back to all parent nodes, backtracking along the same path used in the selection step.

**Figure 1:**
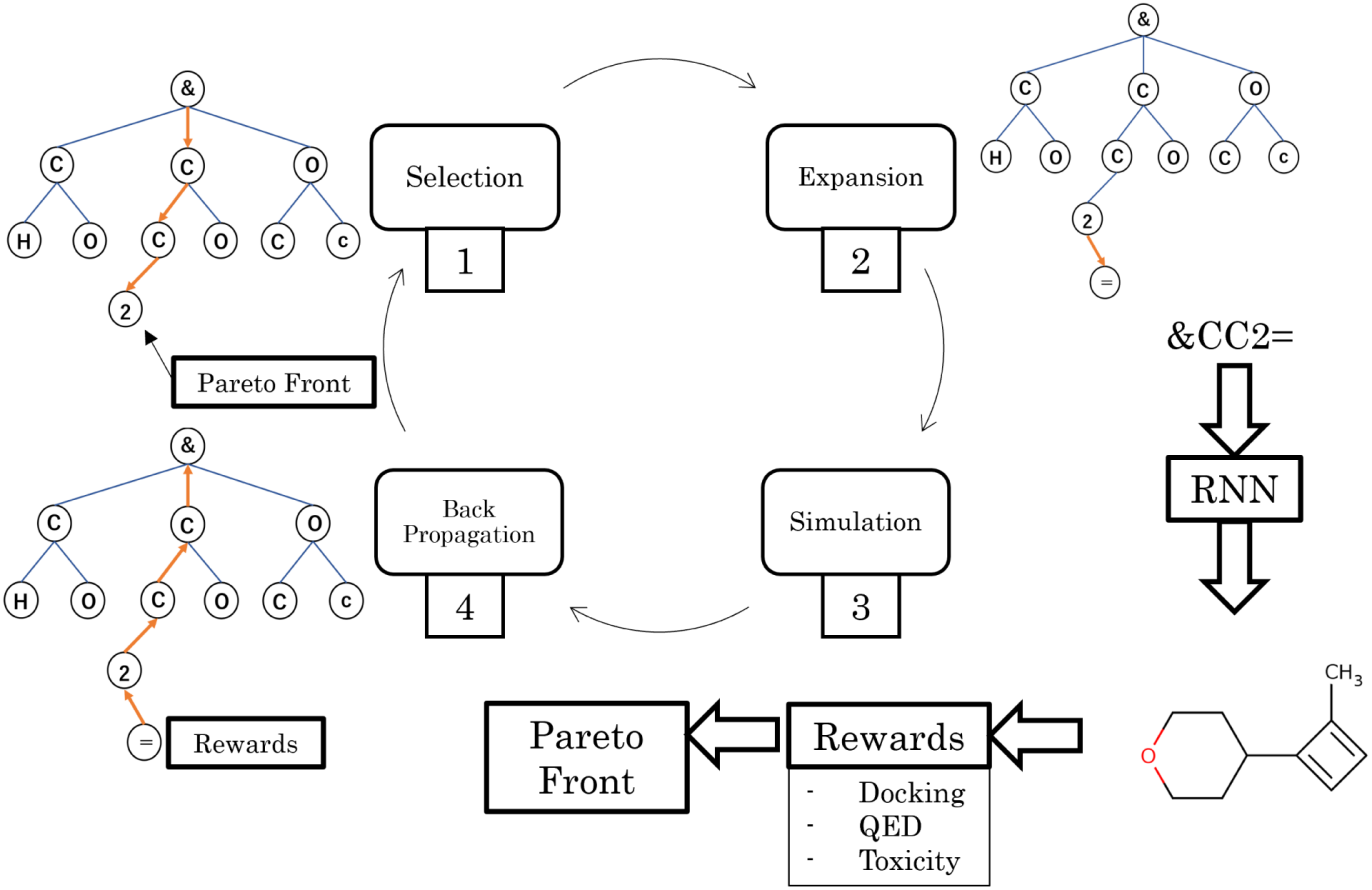
Workflow of Mothra. Subfigures located near the step diagram show the contents of each step. A node in a search tree corresponds to a SMILES character. This workflow consists of four steps: selection, expansion, simulation, and backpropagation. (I) Selection step: Choose a leaf node considering the current Pareto front. (II) Expansion step: Add a child node to a selected node. (III) Simulation step: Complete the substrings of molecules and evaluate their rewards. In addition, update the Pareto front. (IV) Backpropagation step: Feedback rewards to the nodes on the path.

The workflow of Mothra is illustrated in Figure 1. This workflow was derived from the MCTS. Mothra starts with a search tree having only a root node. The root node corresponds to the starting token. The starting token represents the beginning SMILES strings. In the following steps, leaves are added to the search tree. In particular, the following two points have been changed: (i) A reward used to backpropagate is not a scalar value but a multidimensional vector. This change affects the simulation and backpropagation steps. (ii) To consider the Pareto front, the Pareto front engine is applied in the simulation step. The selection step uses the calculated Pareto front to choose a leaf node.

### Pareto Front

Mothra adopts MOO. In MOO, Pareto dominance describes the relationship between two solution points in reward space. Given two points and their reward vectors  and , point *x* is Pareto dominant over *y* if *r*_*xi*_ is not less than *r*_*yi*_ for *i* = 1··· *d*.If a point is not dominated by any other point, it is defined as a nondominated point. A Pareto front is the set of nondominated points (as shown in eq 1).
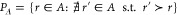
1where *A* is a set of reward vectors. *P*_*A*_ is referred to as the Pareto front in *A*.

**Figure 2:**
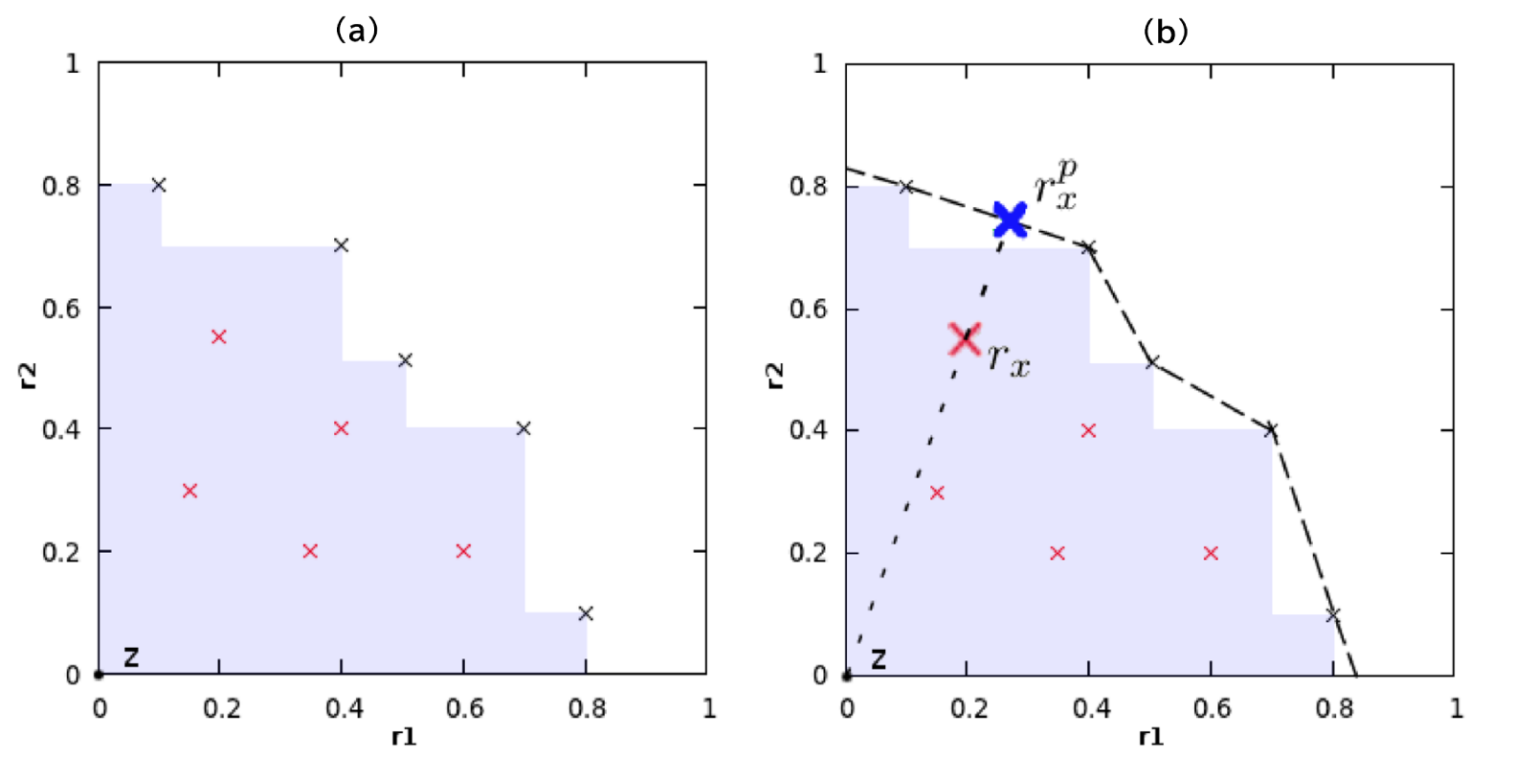
Hyper-volume in a two-dimensional reward space, where the reference point *z* is the original point, and the purple part is the hyper-volume. (a) The X marks represent points belonging to the Pareto front. Black indicates points that contribute to expanding the hyper-volume, and red indicates points that do not. (b) The perspective projection on the Pareto front.^33^

There is no natural priority of the points in the Pareto front. Still, the hyper-volume indicator can impose an order on the set of points,^50^ which is illustrated on a two-dimensional reward space in Figure 2 and defined as eq 2.
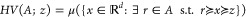
2In eq 2, *z* is a reference point, and μ is the Lebesgue measure on .

One approach to transforming a multiobjective problem into a single-objective problem is the linear combination of all objectives. To avoid setting weights in the linear combination, another approach is to use the contribution to the hyper-volume of issues as a single reward called MOMCTS.^33^ In MOMCTS, the points that maximize the hyper-volume are from the Pareto front. In the selection step, nodes that belong to the Pareto front but do not contribute to expanding the hyper-volume are penalized according to the distance between the point and the projected point on the Pareto front, illustrated in Figure 2.

### Pareto MCTS

The MCTS requires an objective function. For a multiobjective search, the objective function can be set in two ways. One is the linear combination of multiple objective functions, which results in a scalar value. However, this method retains the weight-setting problem. The other method is to use Pareto MOMCTS.^33^ There are two major differences between the Pareto MCTS and MCTS: multidimensional reward vectors and Pareto front calculation. One solution for the latter is NSGA-II^49^ implemented in pygmo, the Python version of pagmo.^48^ The multidimensional reward vector affects the selection step.

On selection nodes in the selection step, nodes in the Pareto front should be sorted in some order via short calculation. One approach to sorting the dominant points is called Pareto rank. This method sorts each point into different layers, like the Pareto front. Nodes in the same layer are nondominant relative to each other. This option requires the maintenance of all nodes, which is too computationally expensive. Instead, this research uses a hypervolume indicator with a projected distance penalty to rank Pareto front nodes. The upper confidence bound (UCB)  controls the balance between exploitation and exploration. The score is estimated using the cumulative reward. The cumulative reward score is calculated using the node visit state. The cumulative reward is defined in eq 3.
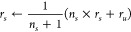
3

In eq 3, *r*_*u*_ is a reward of a new evaluation, and *n*_*s*_ is the number of visits of state *s*.

UCB  is defined in eq 4.
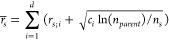
4In eq 4, *c*_*i*_ is the exploration vs exploitation parameter for the i-th component of the reward vector.

An upper bound *U*(*s*) using the hyper-volume (*HV*) indicator of *r*_*s*_ with Pareto front *P* is given in eq 5.
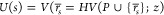
5In eq 5, where *z* is the reference point of the hyper-volume indicator, *U*(*s*) provides a scalar evaluation of a node *s*. However, it maintains a constant value if any point in the Pareto front dominates .

Although it would be sufficient to calculate a hypervolume indicator at all points, it would be very computationally expensive; therefore, Mothra used the projection distance penalty, which can be calculated more quickly. *U*(*s*) is updated to *W*(*s*).
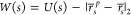
6In eq 6,  is a projection of  onto the upper bound of the Pareto front.

The pseudocode for Mothra is described in Algorithm 1.

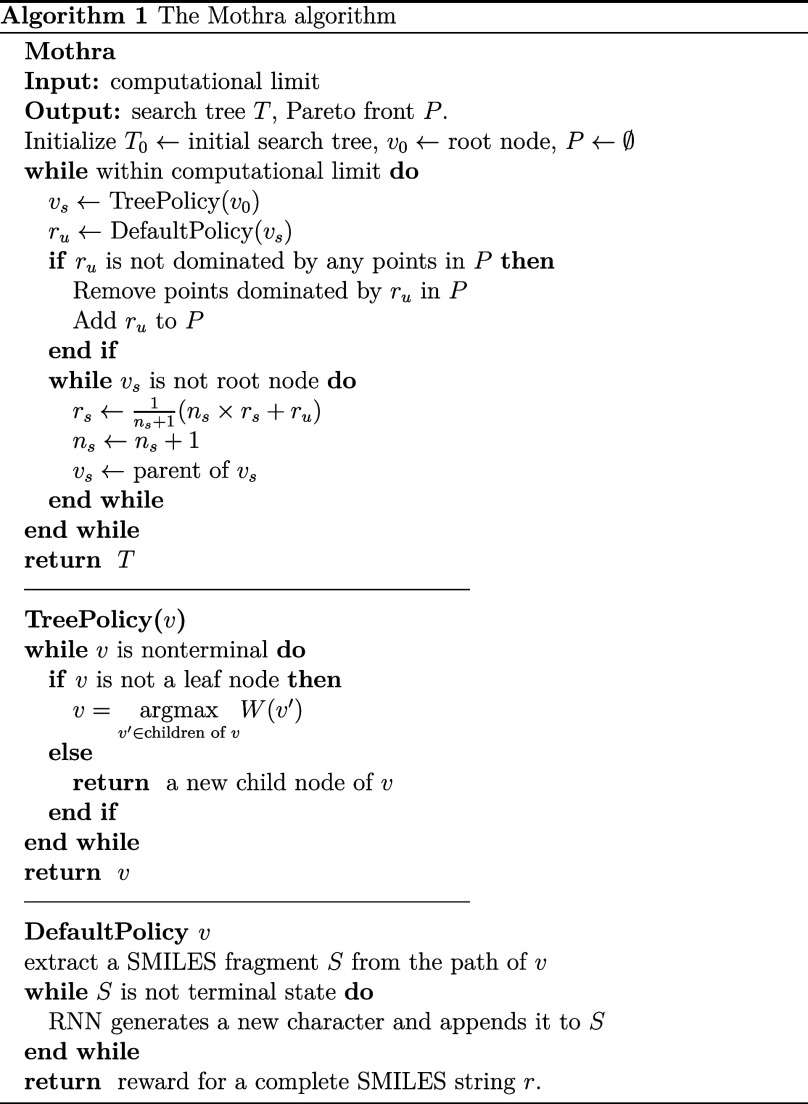



### RNN Training

Mothra uses the *de novo* molecular structure generator ChemTS. Similarly, RNN consists of an 81-dimensional embedding layer and two 256-dimensional GRU layers. The hyperboric tangent activation function is used. In this research, an RNN model was used as a ligand generator. The RNN model was pretrained and remained identical during the ligand search process.

The training of RNN was conducted using the Adam optimizer and the following parameters: a learning rate of 0.01, a batch size of 256, and a total of 100 epochs.

### Data Set

The data set for RNN training was obtained from ZINC.^51^ ZINC is a free public database for ligand discovery that includes more than 20 million molecules in biologically relevant representations. This research used randomly selected approximately 250,000 randomly selected ligand-like molecules represented in SMILES. The data set is the same as that of ChemTS. The ZINC data set could consider the vast chemical space beyond existing drug-like compounds. The data set is provided on the GitHub repository of ChemTS.^52^

3D structural data of the proteins for ligand generation were obtained from the Protein Data Bank (PDB). A kinase (the discoidin domain receptor type 1 (DDR1) kinase) (PDB ID: 3ZOS)^53^ is present. Table 1 lists the SMILES vocabulary used in this study. This vocabulary includes “&” and “\n” as start and end symbols, respectively.

**Table 1:** SMILES Vocabulary

	SMILES description
Atom	C,c,o,O,N,F,n,S,s,Br,I,P
Bonds	- = # $:/\
Functional group	[C@@H], [O-], [C@H], [NH+], [C@], [nH],
	[NH+2], [C@@], [N+], [nH+], [S@], [*N*-],
	[*n*-], [OH+], [NH-], [P@@H], [P@@], [PH2],
	[o+], [CH2-], [CH-], [SH+], [O+],
	[S-], [S+], [S@@+], [NH3+], [n+].
	[S@@], [P@], [P+], [PH], [s+], [PH+]
Terminator	\n

### Objective Functions

*De novo* drug design attempts to create structurally novel lead compounds with desired properties, such as affinity with the target protein, solubility, and membrane permeability. In this research, the docking score, QED, and toxicity probability were set as objective functions.

The docking score evaluates the binding energy between molecules and the target protein. The lower the binding energy is, the better the binding of the molecule to the target protein. The same is true of the docking score on the binding energy. However, when applied to the reward, the higher the reward calculated from the docking score is, the better it could be. The reward function of the docking score is shown in eq 7
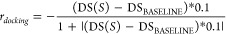
7

In eq 7, DS(*S*) represents the current docking score, and DSbaseline represents the base score for each protein; in this research, it is 0. This function was inspired by SBMolGen.^26^ This function is monotone-increasing. Thus, this function does not sort the docking scores.

The QED^27^ evaluates the drug-likeness of generated molecules on a scale from 0 to 1. The higher the score, the more likely the molecule will become a drug. The reward function of the QED score is as follows (eq 8).
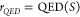
8

The eToxPred^45^ system estimates the toxicity probability of generated molecules on a scale from 0 to 1. The higher the score, the more harmful it is for people to take the molecule. The reward function of the toxicity probability is shown below (eq 9).
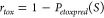
9where *P*_*etoxpred*_ is the toxicity probability estimated using the eToxPred system. The prediction model for the eToxPred system was retrained using the eToxPred-provided data set for compatibility.

The SAscore^25^ evaluates the synthesis accessibility of generated molecules on a scale from 1 to 10. A better SAscore represents a more difficult synthesis. This research uses the SAscore as a filter instead of the final evaluation. The threshold value for the SAscore was 3.5.

### Docking Simulations

To evaluate the binding affinity toward the targeted protein, Mothra opted for AutoDock Vina.^46^ After the protein structure was obtained from PDB, hydrogen atoms were added to the structure via AutoDock tools. The binding pocket of the targeted protein was defined as the rectangular prism set up to completely cover the ligand, starting from the same center as the ligand registered in the PDB. “Exhaustiveness” in the AutoDock Vina options was set to 1 because Mothra must perform docking simulations as many times as the number of compounds generated. The 3D conformations of the generated molecules were transformed by Open Babel^54^ Open Babel, which generated a single conformer or isomer per ligand by selecting the lowest energy.

### Main Search Setting

Before the main search is executed using MOMCTS, the RNN generator must be trained. In the main search, Mothra searched for 14 days on a computer including two Intel Xeon E5–2680 V4 processors and four NVIDIA Tesla P100 GPUs. A tool that selects molecules by considering a searched chemical space is available on the same GitHub link running on the Docker system.

### Metrics

To assess the quality of Mothra as a molecular optimizer, the following metrics, which are often used in evaluating molecular generative models were calculated. The duplication ratio is the rate at which the generated molecules are the same (even if their SMILES representations differ). The novelty^55^ is the ratio of valid and unique molecules absent in the training data set, provided in ChemTS in this case. The internal diversity is the mean over the Tanimoto similarity between the generated molecules.^56^ The uniqueness is the ratio of the number of valid and unique molecules.

## Results

In this experiment, we validated the performance of Mothra’s multiobjective molecular generation using DDR1 kinase (PDB ID: 3ZOS) as the target protein. Potatinib, the cocrystal ligand of 3ZOS, revealed a docking score of −9.4 kcal/mol.

**Figure 3:**
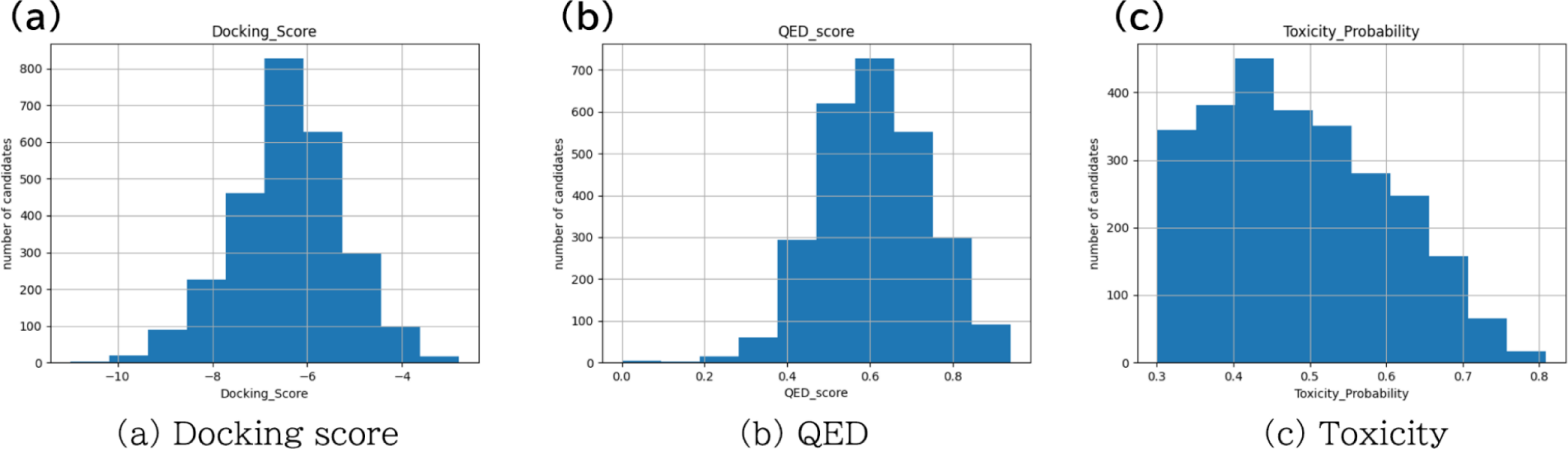
Population of molecules produced for each metric for compound generation by targeting DDR1 kinase (PDBID: 3ZOS). Figure (a), (b), and (c) show the population of molecules in Docking score, QED, and toxicity probability, respectively.

Figure 3 shows the distribution of generated molecules in each objective function. In the following figures, because the docking score axis shows raw values, lower values indicate stronger binding. Each distribution on each axis is a single-peak distribution with broad bases on both sides of the peak; therefore, Mothra accomplished a broad search.

**Figure 4:**
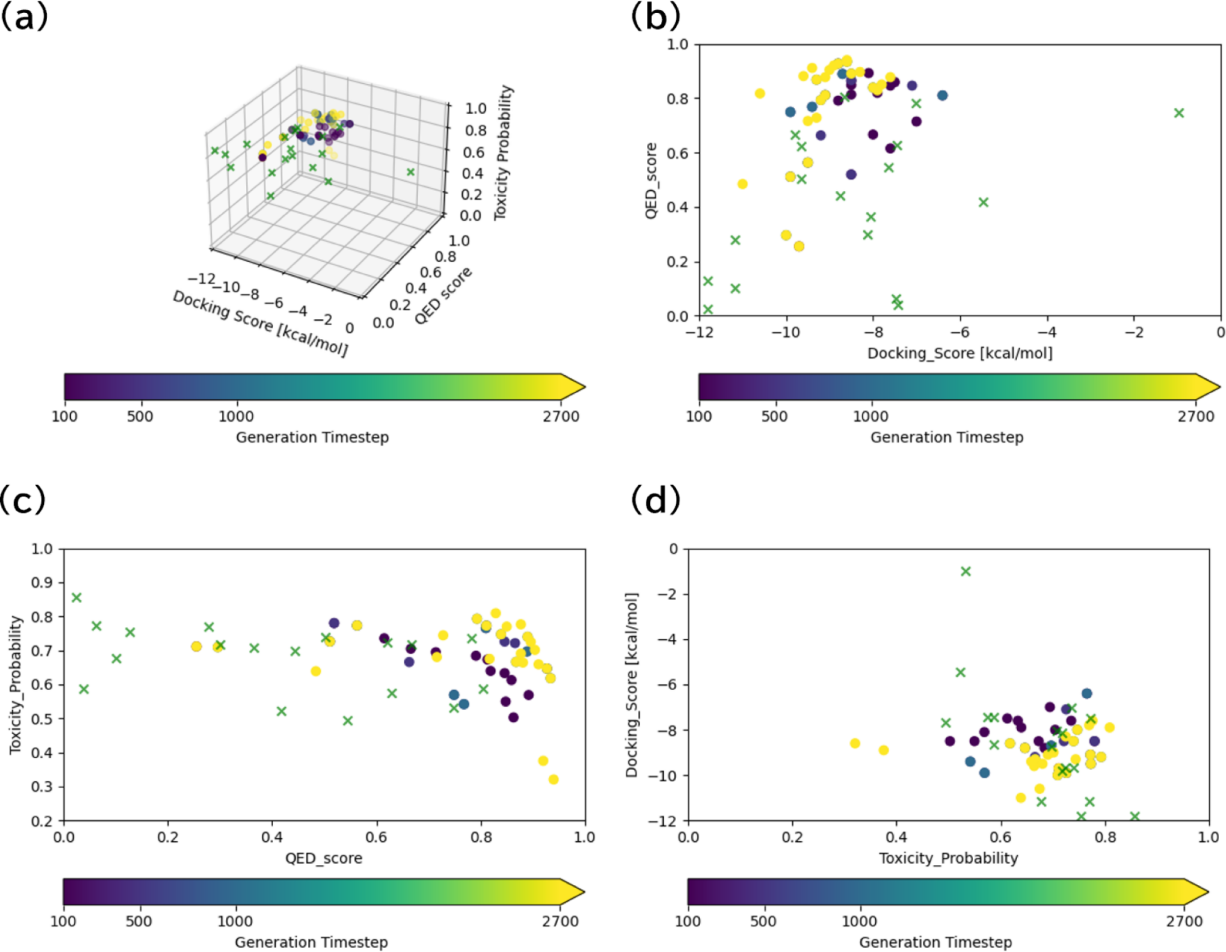
Scatter plot of Pareto front. Figures (b),(c), and (d) draw the relevance between the docking score and QED, QED and the toxicity probability, and toxicity probability and the docking score, respectively. Green crosses correspond to known molecules binding to the target protein registered in the ChEMBL database. The colors of dots correspond to timesteps. Pareto fronts were calculated when 100, 500, 1000, and 2664 molecules were generated.

Figure 4a shows the docking score, QED, and toxicity probability of the optimized compounds for DDR1 kinase. Figure 4b–d shows the two-dimensional point clouds obtained by projecting the three-dimensional point cloud shown in Figure 4a onto the plane of the two objective functions. As time passed, a search was carried out to increase the HV, resulting in better molecules. Some generated molecules are optimized beyond known ligands registered in the ChEMBL database. However, even in the Pareto front, some molecules have a low value in one index despite having high values in others. These molecules are considered to be weak Pareto optimal solutions. Such solutions are inevitably included during the calculation of the Pareto front; however, they can easily be removed by filtering after generation. All of the SMILES-validly generated molecules are reported in the Supporting Information.

**Figure 5:**
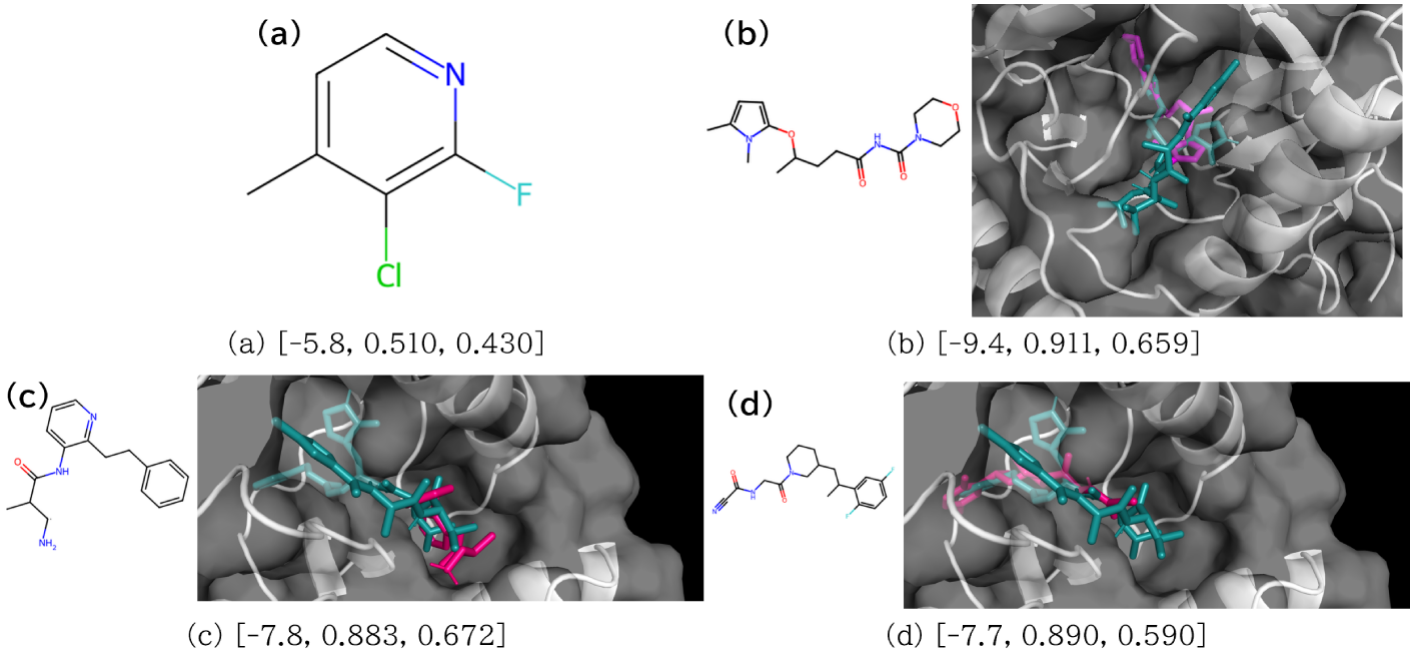
Generated molecules with DDR1 kinase and their docking poses. Figure (a) shows the first generated molecule and Figure (b) to (d) show molecules in the last Pareto front with DDR1 kinase and their docking poses. The list [A, B, C] shows the evaluation score on each caption. A corresponds to the docking score [kcal/mol], B to the QED score, and C to the toxicity probability.

After filtering, we selected some drug candidates from the generated molecules. Figure 5 show generated molecular structures with their objective function values and docking poses. The figures show that Mothra accurately generated molecules in the optimal direction. These experiments were performed with the objective function of binding affinity, so these molecules were appropriately bound to the pocket in the targeted protein surfaces. Comparing Figure 5b to Figure 5c, the docking score and QED were improved, but the toxicity probability was maintained. This result indicates that Mothra captured the trade-off relationship by lying on the objective functions.

The percentage of duplicates was . The novelty is . The other metrics are shown in Table 2. From the result in the Internal Diversity, Mothra generated diverse molecules.

**Table 2:** Metrics for Assessing the Molecular Generative Models^a^^b^^c^

Method	PDBID	Validity	Uniqueness	Internal Diversity
MOO–DENOVO^39^	3B7E	0.995	**0.986**	0.733
DeLA-DrugSelf^41^	6KPC	**1.000**	0.802	0.84
Mothra	**3ZOS**	0.735 ± 0.00496	0.976 ± 0.00712	**0.886** ± **0.00142**

a The data of existing methods are referred to in each paper.

b The PDBID column shows the PDBIDs of the target protein.

c Mothra was run five times, so the results are shown in both means and s.d.s.

**Figure 6:**
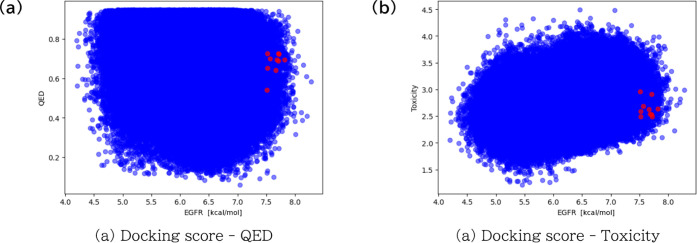
Relevance of the docking score with EGFR (target) protein and QED or toxicity index. Red points show the top 10 molecules in terms of DScore. Blue points show the other molecules.

To evaluate Mothra, we experimented with ChemTSv2.^31^ Following this study, we set EGFR as the target protein. The other parameters were ERBB2, Abelson tyrosine-protein kinase, proto-oncogene tyrosine-protein kinase, lymphocyte-specific tyrosine-protein kinase, platelet-derived growth factor receptor beta, vascular endothelial growth factor receptor 2, and ephrin type-B receptor 4, set as low-affinity proteins. Additionally, we developed solubility, permeability, metabolic stability, SAscore, and QED as objective functions to be maximized, as well as toxicity as a function to minimize. We made a configuration file based on the template file provided in the GitHub repository.^57^ We changed the duration time to 336 h (14 days) in the “setting_dscore.yaml” file because we observed the molecules that were generated after running for 14 days (Figure 6). The experiment was run on a computer with one Intel Xeon Gold 5318Y CPU and one NVIDIA RTX 4090 GPU. From the plots, ChemTSv2 did not correctly capture the Pareto front, dropping from the estimated Pareto front. Furthermore, ChemTSv2 did not suggest the Pareto front but suggested molecules following the DScore reward. ChemTSv2 hid the trade-off relationship in suitable molecules and may lead to a misinterpretation of the results. Therefore, Mothra is a superior method.

## Discussion

**Figure 7:**
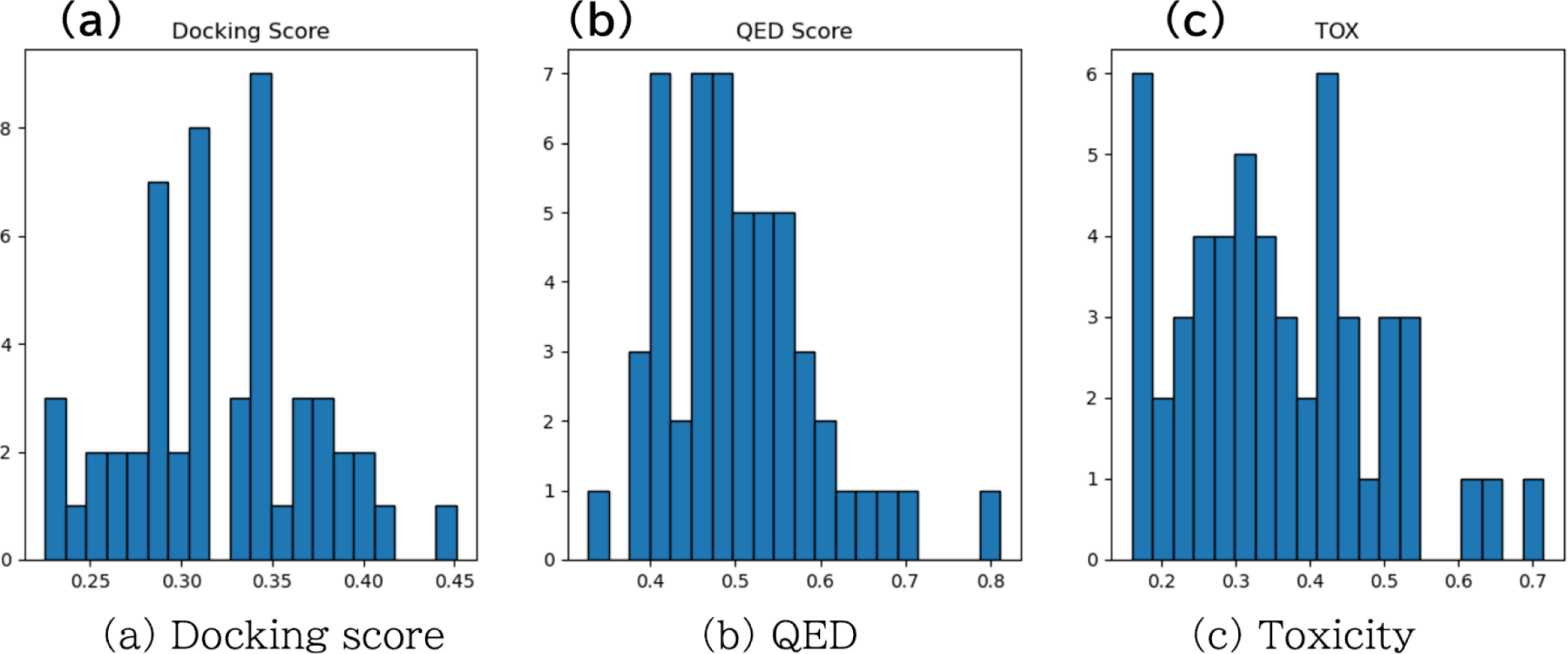
Distribution of compounds generated by single-objective optimization. The docking score toward DDR1 kinase was used as the objective function.

**Figure 8:**
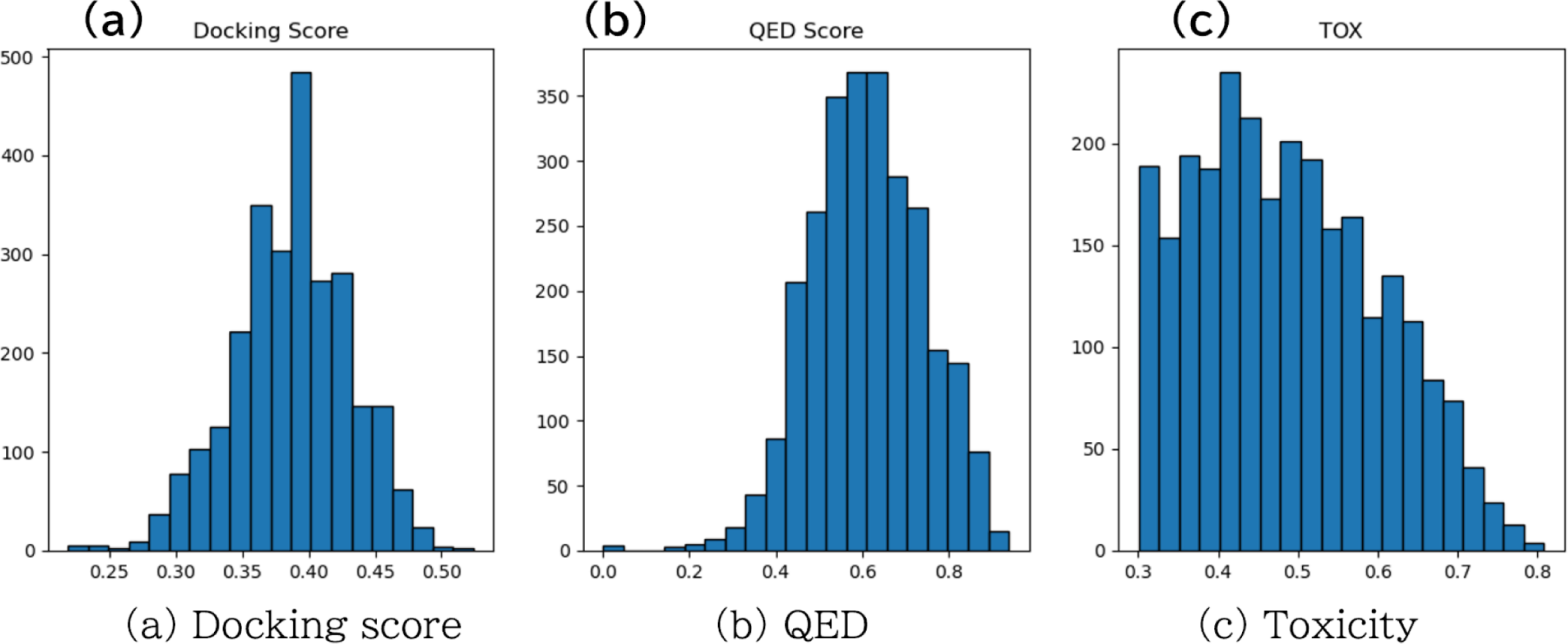
Distribution of compounds generated by MOO. The docking score toward DDR1 kinase, QED, and toxicity probability were used as the objective function.

To verify the effectiveness of MOO, we compared the distribution of compounds generated by single-objective and MOO. For compatibility with existing code, especially in modules for the Pareto front calculation engine, we set the affinity and constant function as the objective functions in the single-objective optimization, and the affinity, QED, and toxicity probability as the objective functions in the MOO, as in the experiment. We ran five 14-day experiments. We show the result of one experiment. Figures 7 and 8 show the distribution of each objective function. In single-objective optimization (SOO), the compounds improved only in affinity but remained low in the other indices, viz., QED and toxicity probability. However, in MOO, good compounds were generated in terms of each index. In all experiments, the number of generated molecules was about 100. The less comes from the character of MCTS. The MCTS digs the local minima straightly. In SOO, other leaves in the search tree might not be chosen because obtaining a high reward in the binding affinity is difficult in the small ligands. In MOO, however, MOMCTS could consider other objectives in every search step. Therefore, MOO can generate compounds with the desired properties in drug discovery scenarios more effectively than single-objective optimization.

It is essential to note the number of objective functions. Because this is an MOO, there is no theoretical limit to the number of objective functions that can be used simultaneously. However, if the number becomes too large, we cannot efficiently search a vast chemical space. Optimization problems with four or more objective functions are called “many-objective” problems because of their complexity.^58^ To avoid this problem, limitations on the properties of molecules are imposed after the generation system. Using a threshold, the generation system keeps its performance, and the probability of obtaining the desired molecules is higher.

As shown by the above results, Mothra successfully generated molecules in the desired direction in the multiobjective optimization. Most of the generated molecules were optimized beyond the first generated molecule for the target protein.

**Figure 9:**
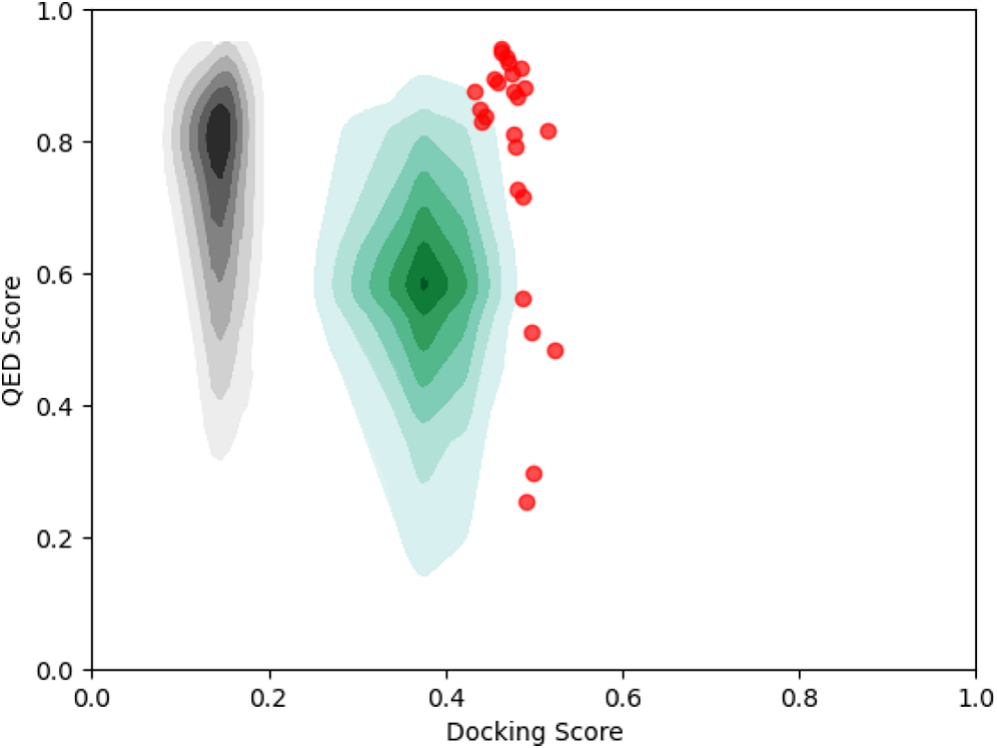
Distribution of compounds. The black and green hills indicate the distributions of ZINC and all generated molecules, respectively. The red dots indicate the molecules belonging to the Pareto front. The Docking Score is normalized (larger is better).

It is worthwhile to briefly consider the differences between the general distribution of molecules, the so-called chemical space, and the generated molecules. Figure 9 shows the differences between the two by overlaying images. Though the generator of Mothra was trained in the distribution of the ZINC (black), Mothra generated in the different molecular distributions. Mothra can optimize molecules with Pareto MOMCTS over a fixed RNN generator. Because Mothra starts with a single root node, all generated molecules are found by RNN and MOMCTS. Furthermore, the RNN was a fixed generator. MOMCTS finds desired molecules from the general chemical space with the information on having generated molecules. Using Mothra, the user can search for compounds with the desired properties in the chemical space.

The RNN generator learns the grammar of SMILES, not the entire structure of the compound. For chiral carbons, we observed cases in which absolute configurations were explicitly generated, and cases in which they were not. In addition, the RNN sometimes described meaningless absolute configurations by adding “@” to achiral carbon atoms. Therefore, all compounds in Figure 5 are shown without the absolute configuration. However, transformers, which are deep neural network generative models that are larger than RNNs, have difficulty recognizing chirality in molecules.^59^ Thus, changing the generator to use something other than RNNs or transformers is an issue for the future.

## Conclusion

In this study, we developed a multiobjective molecular generation system, Mothra, that simultaneously optimizes multiple properties using the Pareto optimization. Our method is intended for use in the initial steps of drug discovery. Thus, only objective functions and target proteins were set. Our method provided the desired molecules. In this experiment, there were three objective functions and a single constraint: affinity(docking score), drug-likeness, and toxicity, as well as synthetic accessibility. To evaluate our method, a protein was set as a target, and the generated molecules were checked in the chemical space. The generation results were acceptable, based on the distribution of the molecules. Compared to a single-objective molecular generation system, Mothra generated molecules in the desired direction using multiple objective functions. Furthermore, Mothra captured the Pareto frontier of the reward space while generating molecules. The previous multiobjective molecular generative model could not handle the Pareto optimization because its objective function is a linear combination of all objective functions. These results indicate that Mothra could be applied in practical drug discovery. After structural information about target proteins is obtained, Mothra can generate seeds for drug discovery. To enhance the practical usability of Mothra, it would be desirable to allow users to define objective functions according to their specific needs flexibly.

There are still limitations to this method. In MOO, there is no restriction on the number of functions used for evaluation, but there are practical problems. In this study, we set three evaluation functions and optimized them simultaneously, but there may be more than 50 compounds that belong to the Pareto front. The population of Pareto frontiers causes problems extending the solution space and resulting in longer search time. Furthermore, increasing the number of evaluation functions increases the number of things that can be considered, but increases the number of compounds that belong to the Pareto front, making it difficult to proceed with the search. On the other hand, if the number of evaluation functions is reduced, the number of compounds belonging to the Pareto front will be shrunk, but evaluation items that should be considered in drug discovery cannot be considered. When increasing the number of evaluation functions, it is also necessary to consider the correlation between evaluation functions. When considering the mapping of compound space, each axis should be independent. Therefore, the absolute value of the correlation coefficient between evaluation functions should be closer to 0. The correlation coefficient between QED and toxicity using ChEMBL^60^ as a population was 0.24, so those two evaluation functions were adopted. When setting more evaluation functions than this, introducing an evaluation function with a low correlation for both QED and toxicity has the advantage of being able to consider more evaluations than the cost of having a large number of evaluation functions.

## Data Availability

The source code and pretrained model for Mothra, developed in this study, along with the Docker image for the Pareto front explorer and compound picker, designed to streamline the selection and visualization of optimal chemical compounds, are released under the GNU General Public License v3.0 and available at https://github.com/sekijima-lab/Mothra.

## Supporting Information Available

All of the SMILES-validly generated molecules by Mothra (TXT)
